# A three‐dimensional placoderm (stem‐group gnathostome) pharyngeal skeleton and its implications for primitive gnathostome pharyngeal architecture

**DOI:** 10.1002/jmor.20706

**Published:** 2017-05-23

**Authors:** Martin D. Brazeau, Matt Friedman, Anna Jerve, Robert C. Atwood

**Affiliations:** ^1^ Department of Life Sciences Imperial College London, Silwood Park Campus Buckhurst Road SL5 7PY United Kingdom; ^2^ Department of Earth Sciences Natural History Museum London SW7 5BD United Kingdom; ^3^ Museum of Paleontology and Department of Earth and Environmental Sciences University of Michigan 1109 Geddes Ave Ann Arbor Michigan 48109‐1079; ^4^ Beamline I12‐JEEP, Diamond Light Source, Harwell Science and Innovation Campus Didcot Oxfordshire UK OX11 0DE

**Keywords:** branchial skeleton, Devonian, hyoid arch, Hunsrück Slate, *Paraplesiobatis*, synchrotron tomography

## Abstract

The pharyngeal skeleton is a key vertebrate anatomical system in debates on the origin of jaws and gnathostome (jawed vertebrate) feeding. Furthermore, it offers considerable potential as a source of phylogenetic data. Well‐preserved examples of pharyngeal skeletons from stem‐group gnathostomes remain poorly known. Here, we describe an articulated, nearly complete pharyngeal skeleton in an Early Devonian placoderm fish, *Paraplesiobatis heinrichsi* Broili, from Hunsrück Slate of Germany. Using synchrotron light tomography, we resolve and reconstruct the three‐dimensional gill arch architecture of *Paraplesiobatis* and compare it with other gnathostomes. The preserved pharyngeal skeleton comprises elements of the hyoid arch (probable ceratohyal) and a series of branchial arches. Limited resolution in the tomography scan causes some uncertainty in interpreting the exact number of arches preserved. However, at least four branchial arches are present. The final and penultimate arches are connected as in osteichthyans. A single median basihyal is present as in chondrichthyans. No dorsal (epibranchial or pharyngobranchial) elements are observed. The structure of the pharyngeal skeleton of *Paraplesiobatis* agrees well with *Pseudopetalichthys* from the same deposit, allowing an alternative interpretation of the latter taxon. The phylogenetic significance of *Paraplesiobatis* is considered. A median basihyal is likely an ancestral gnathostome character, probably with some connection to both the hyoid and the first branchial arch pair. Unpaired basibranchial bones may be independently derived in chondrichthyans and osteichthyans.

## 1 INTRODUCTION

The pharyngeal skeleton has long been implicated in theories of the origin of jaws and is a key system in understanding the early diversification of feeding and respiratory systems in jawed vertebrates (gnathostomes). This skeletal system consists of mandibular and hyoid arches; and the branchial skeleton, or gill arches. It comprises a jointed network of bones and cartilages surrounding the mouth and pharynx (throat). Each arch consists of a chain of elements extending from a dorsal origin on either the braincase or vertebrae, and extending around the throat and meeting at the ventral midline, where one or more medial (or paired) corpuses may connect one or more arches.

The gill skeleton, in particular, as well as its musculature have classically provided a rich source of character data for studies of gnathostome interrelationships (Nelson, 1969; Pradel, Maisey, Tafforeau, Mapes, & Mallatt, 2014; Wiley, 1979). Unfortunately, fossil examples of complete gill skeletons from early gnathostomes are rare owing to their typically weak mineralization and deep anatomical position that often makes them inaccessible to study. A lack of information from stem‐group gnathostomes leaves considerable uncertainty about pharyngeal skeleton character polarities, limiting their phylogenetic usefulness.

The two main divisions of the gnathostome crown group (Osteichthyes and Chondrichthyes) differ significantly in the pattern of topological relationships of their pharyngeal arch segments, particularly in the ventral elements. Both chondrichthyans and osteichthyans possess one or more median basibranchial elements. Chondrichthyan hyoid arches consist of ceratohyals attaching directly to a median hypohyal element (Allis, 1922, 1923; Carvalho, Bockmann, & de Carvalho, 2013; Garman, 1913; Pradel et al., 2014; Shirai, 1992). By contrast, in osteichthyans, the ceratohyals attach to a median bone (usually termed a basibranchial) via hypohyals, (Allis, 1897, 1922; Grande & Bemis, 1998; Jarvik, 1954, 1972) the latter of which are usually considered absent in chondrichthyans. In both chondrichthyans and osteichthyans, each branchial arch terminates ventrally and medially by a hypobranchial bone or cartilage, which may connect either at the midline to its antimere or to a ventral basibranchial bone or cartilage. In osteichthyans, the hypobranchials are considered to be “anteriorly directed” and at least one anterior branchial arch pair usually joins the same basibranchial as the hyoid arch. In elasmobranch chondrichthyans, all but the first hypobranchials are (usually) posteriorly directed, and join either at the anatomical mid‐line or to a basibranchial copula. The first hypobranchial is often anteriorly directed and connected to the posterolateral angle of the basihyal (see e.g., Garman, 1913).

All of these contrasts are potentially phylogenetically informative variables (Brazeau & Friedman, 2014; Pradel et al., 2014). Although consideration of fossils aids the separation of plesiomorphic and derived conditions (Pradel et al., 2014), considerable uncertainty remains. The lack of an outgroup information from stem‐group gnathostomes has inhibited character mapping exercises attempting to reconstruct primitive branchial arch conditions (Pradel et al., 2014).

Fossilized gill skeletons are extremely poorly known from jawless gnathostome outgroups (Conway Morris, & Caron, 2014; Janvier & Arsenault, 2007; Janvier, Desbiens, Willett, & Arsenault, 2006). However, partially ossified examples exist in the placoderms: an assemblage of Paleozoic jaw‐bearing stem‐group gnathostomes. Unfortunately, these gill skeletons are weakly mineralized and therefore tend to be poorly preserved. Complete, articulated placoderm fossils, particularly with preserved endoskeletons, are extremely rare. However, multiple examples are known from the exceptional Early Devonian Hunsrück Slate *Lagerstätte* of Germany. Among these are the anatomically and phylogenetically enigmatic “stensioellids,” including *Stensioella*, *Pseudopetalichthys*, *Nessariostoma*, and *Paraplesiobatis*. This assemblage is so morphologically diverse, however, that the group is unlikely to be phylogenetically coherent. Nevertheless, these taxa are known from complete and articulated fossils. Furthermore, these fossils exhibit X‐ray contrast (Gross, 1962) and are therefore amenable to computed tomography (CT) investigations.

This article provides further details on the morphology of placoderm gill arches through a synchrotron CT‐analysis of *Paraplesiobatis heinrichsi* Broili (1933). Data from *Paraplesiobatis* confirm some generalized aspects of placoderm branchial arch anatomy observed in less complete examples. However, it also presents peculiarities that raise questions about the homology of some elements in chondrichthyan and osteichthyan branchial skeletons. A comparative analysis of basal gill arch elements in early and modern gnathostomes is here used to infer some aspects of primitive branchial arch patterns.

## 2 MATERIALS AND METHODS

### 2.1 Specimen

This investigation is based on the type specimen of *Paraplesiobatis heinrichsi* Broili (1933) from the Schlossparkmusuem (Karl‐Geib‐Museum), Bad Kreuznach KGM 1983/294 (assigned by Wuttke, 1986). The specimen is a complete, articulated dermal armor and trunk squamation. It has previously been described by Broili (1933) and by Gross (1962).

### 2.2 Geological context

The Hunsrück Slate is an offshore, muddy facies of Early Devonian age in the Rhenish Mountains of southeastern Germany (Bartels, Briggs, & Brassel, 1998; Schindler, Sutcliffe, Bartels, Poschmann, & Wuttke, 2002), but lacks a formal stratigraphic status despite common usage in the paleontological and geological literature. Bartels et al. (1998) suggest that the Hunsrück Slate is effectively comparable to a group, rather than formation, in lithostratigraphic nomenclature. The exact collection horizon for *Paraplesiobatis* within the Hunsrück succession is unclear, but it likely derives from the clay‐rich, mid‐basinal Kaub Formation at the Bundenbach locality (Gross, 1962). An ash layer near the base of the Kaub Formation is radiometrically dated as 407.7 ± 0.7 Ma(Kaufmann, Trapp, Mezger, & Weddige, 2005), while the top of the formation at Bundenbach extends into the *Nowakia elegans* Dacryoconarid Zone (De Baets, Klug, Korn, Bartels, & Poschmann, 2013). The *N. elegans* Zone lies within the *Polygnathus inversus* Conodont Zone, the top of which has been spline‐dated to 397.68 ± 2.144 Ma (Becker, Gradstein, & Hammer, 2012). Thus the age of *Paraplesiobatis* can be roughly constrained to between 398 and 408 Ma.

### 2.3 Synchrotron tomography

The specimen was scanned using synchrotron radiation X‐ray microtomography at the I12‐JEEP beamline of the Diamond Light Source, Didcot, UK (Drakopoulos et al., 2015). Tomography was performed by acquiring X‐ray radiographs at 0.1 deg angular spacing using lambda=0.0124 nm (100 keV) monochromatic X‐rays, illuminating a 0.9 mm Cadmium Tungstate (CdWO4) scintillator which was imaged by microscope optics onto the detector of PCO4000 CMOS camera (PCO‐AG, Germany), with a projected pixel size of 12.6 micron at the sample. The tomographic 3‐d images were reconstructed using filtered back projection and ring suppression (Titarenko, Titarenko, Kyrieleis, Withers, & De Carlo, 2011). The resulting voxel size was 12.7 µm. Because of the size of the specimen, seven overlapping scans were generated using the automated vertical translation stages supporting the tomography rotation stage. These were later “stitched” together in postprocessing.

Tomography **data can be accessed at**: https://figshare.com/s/8bdd8e20f76de18febf9

### 2.4 Segmentation and virtual modeling

The resultant volume was loaded in Mimics (Materialise Software) and completed in version 18 and some earlier versions. Particular care was taken to avoid the influence of a pair of heavy ring artefacts and the “brightness artefacts” caused by highly dense pyrite crystals (Supporting Information Figure 1). Furthermore, the discontinuous grayscale normalization between each separate scan series comprising the total volume (Supporting Information Figure 1) caused difficulties in selecting consistent threshold values. Therefore, most of the individual bones were formed from multiple masks that were later united using Boolean operations. Surface models used in this study are freely available at https://doi.org/10.6084/m9.figshare.4555462

## 3 DESCRIPTION

**Figure 1:**
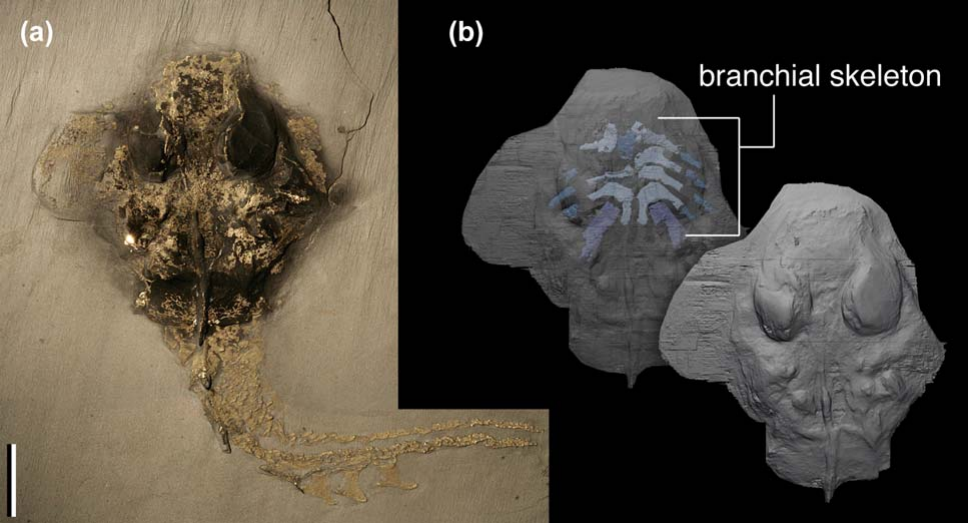
*Paraplesiobatis* specimen KGM 1983/294 (a) and virtual renderings of synchrotron light computed tomography showing solid (lower right) and transparent views (upper left) with branchial arches (b). Scale bar = 1 cm

**Figure 2:**
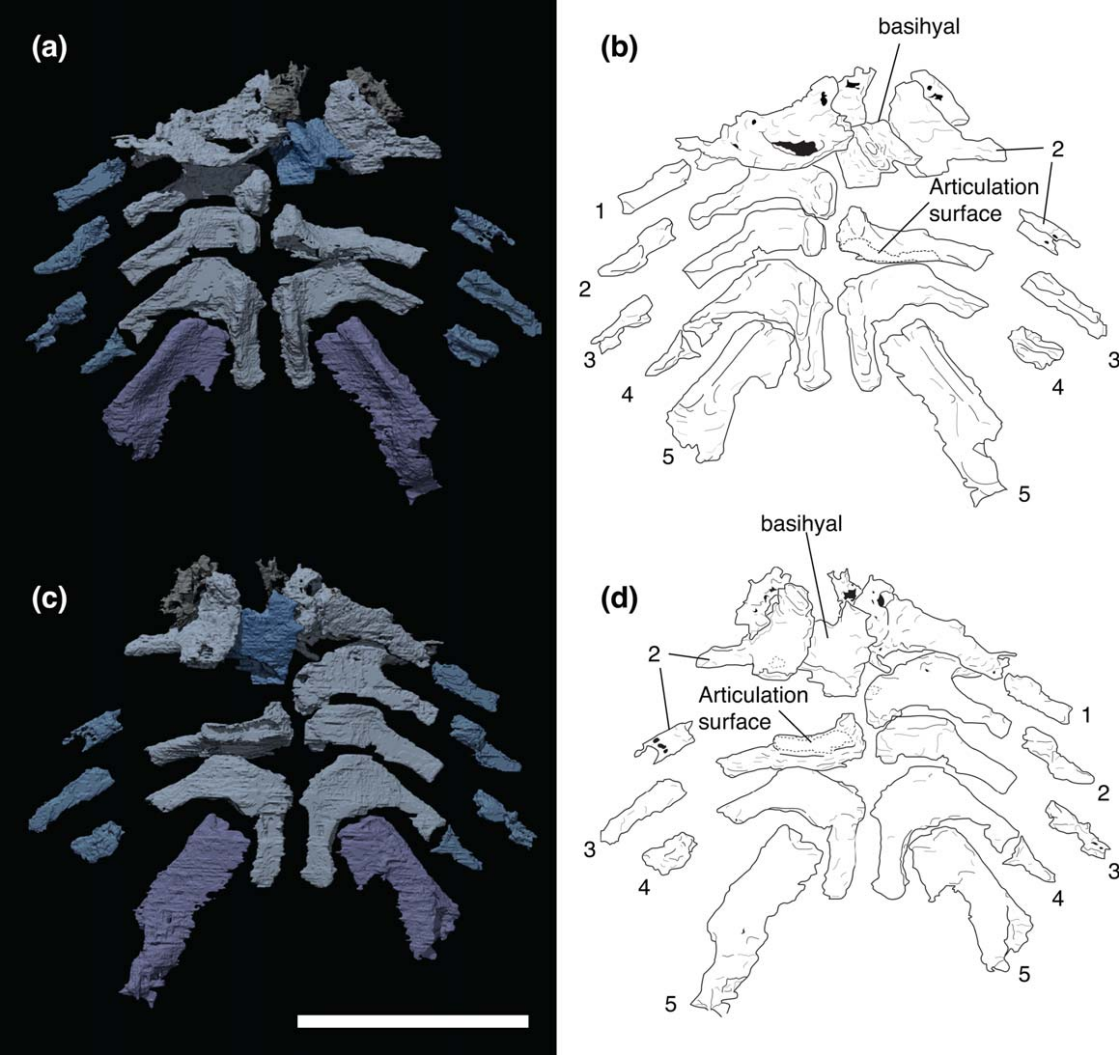
*Paraplesiobatis*, virtual renderings and interpretive illustrations of articulated gill skeleton of KGM 1983/294. Dorsal view (a, b). ventral view (c, d). Numbers indicate individual arch orders referred to in the text, but not necessarily direct homologies to other gnathostomes. Scale bar = 1 cm

The gill skeleton of KGM 1983/294 is preserved as a segmented network of perichondrally ossified bones (Figures 1 and 2). The skeleton is mostly intact and nearly complete ventral gill basket spanning the entire breadth of the skull. No dorsal (epibranchial or pharyngobranchial) elements are preserved. The gill skeleton comprises a single, median basihyal and set of four to five segmented arches (explained below). The first four arches consist of two segments: a medial segment (that joins its antimere across the ventral midline), and a lateral segment. The fifth arch consists of a single visible segment that connects to the posterolateral facet of the fourth arch, rather than meeting its antimere. There are no additional unpaired median bones preserved.

The right side is nearly completely preserved in life position (Figure 2). However, the left, anterior bones are disrupted and displaced, possibly dislodged by sediment or other intrusions to the mouth. As this description will show, there is some uncertainty in interpreting the arch numbers with respect to their serial identities in other gnathostomes. Because of this, the arch numbers assigned here are not necessarily intended to indicate exact identity with other gnathostomes and therefore refer only to the antero‐posterior order of the arches.

The basihyal is roughly trapezoidal in dorsoventral aspect. It has been displaced taphonomically: rotated clockwise about ten degrees from the midline, and pitched upwards by about 30 degrees from horizontal. These angles suggest that it has been pushed posteriorly from its original life position. The anterolateral corners are deeply notched. As in the phyllolepid placoderm *Cowralepis* (Ritchie, 2005) there is a longitudinal keel with a convex profile. Similar to *Cowralepis*, the keel is deepest at its posterior and tapers upwards anteriorly to meet the anterior margin of the bone. The posterior boundaries of the bone are indistinct in the scans, suggesting incomplete mineralization, possibly corresponding to the positions for articulations with other arch elements.

Each of the distal arch elements are short, roughly rectangular bony rods (Figure 2). Most appear featureless owing to the poor scan quality, however some resolve well enough to show a longitudinal ventral ridge. The remaining description focuses only on the medial elements of each arch.

**Figure 3:**
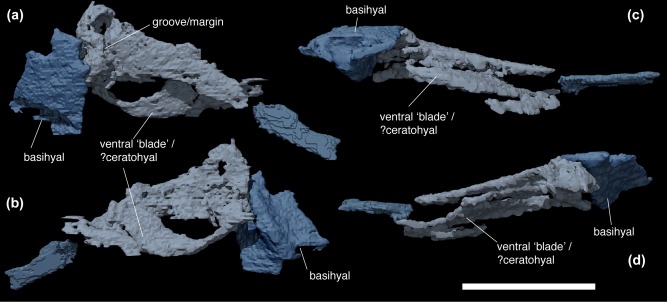
*Paraplesiobatis*, basihyal and first arch elements. Dorsal view (a). Ventral view (b). Posterior view (c). Anterior view (d). Scale bar = 1 cm

The first arch in the series is preserved only on the right side (however, a small fragment of it may be preserved on the left). This is the most problematic element to describe because the tomographic data is severely afflicted by a number of artifacts. The bone has an unusual morphology, consisting of a broad, roughly oval ventral “blade,” and a dorsal flattened rod‐like region (Figure 3). This element is described as a single structure here. However, we consider it possible that it is a composite of two separate bones, a ceratohyal and first branchial arch, that were brought into close proximity when the pharynx was disrupted and cannot be resolved in the scan.

Arches 2 to 4 consist of a posterolaterally swept rod‐like bone (Figure 2). In dorsoventral aspect, their outline consists of a broad medial region that tapers abruptly, at about mid‐length along the anterior margin to form a narrow, posterolaterally angled rod. The dorsal surface is flat and mainly featureless. On the ventral side, the surface bears a “basibranchial” tumidity. The anterior and posterior faces of expanded region appear to have broad areas of articulation with the preceding or following members of its series (Figure 2). The medial elements are flat, and relatively featureless on their dorsal side. However, they have a deep ventral tumidity at their medial extremity, corresponding to the position of the hypobranchials of placoderms in Stensiö's (1969) terminology or the basibranchials of other authors (Carr, Johanson, & Ritchie, 2009; Ritchie, 2005). It is notable that in KGM 1983/294 these are completely continuous with the lateral rod‐like projection (Figure 2). It is, therefore, unclear whether these correspond to fused ceratobranchial, hypobranchial, and basibranchial elements.

The second arch has a strongly arched anterior margin along its medial half in dorsoventral view (Figure 2). It arcs anterolaterally, before sharply turning backwards to narrow into its lateral rod‐like flange. By contrast, the same region of the third arch is roughly parallel‐sided with the posterior margin, giving the medial area a roughly rectangular profile.

The fourth arch differs from the preceding arches in that the tumid portion extends posteriorly as a process nearly as long as the bone's width. The result is a crescent‐shaped bone, opening posterolaterally. The fifth arch articulates (or at least points toward) the crux of the fourth arch medial element (Figure 2).

The fifth arch consists of a single observed bone. The bone is robust and roughly rectangular in dorsoventral profile. In its anterior two thirds, it is deeply keeled along the anterior margin; the posterior margin thinning dorsally to a bladed edge. Distally, the ventral keel flares out to a bulge spanning the width of the bone. This bulge accommodates a deep fossa on the anterolateral face of the bone.

## 4 DISCUSSION

### 4.1 Alternative interpretation of *Pseudopetalichthys*

**Figure 4:**
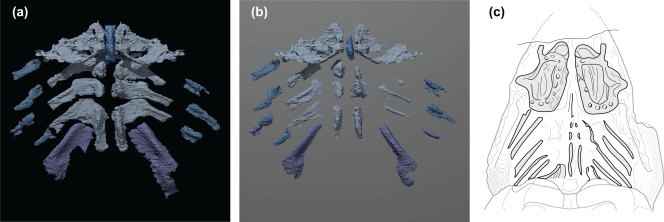
Alternative reconstructions of *Paraplesiobatis* and comparison with *Pseudopetalichthys*. Partially reconstructed gill skeleton with left and right first arch elements mirrored, and basihyal and posterior elements restored to approximate life condition (a). Reconstruction in panel A partially embedded in a virtual plane resulting in apparent segmented pattern (b). Reconstruction of *Pseudopetalichthys* (redrawn from Gross, 1962) with pharyngeal arch elements highlighted to show correspondence with *Paraplesiobatis* suggesting that the segmented appearance may be misleading (c). Not to scale

The most complete placoderm branchial skeleton known belongs to the now lost (Wuttke, 1986) type and only specimen of *Pseudopetalichthys problematicus* Moy–Thomas. It shares in common with *Paraplesiobatis* posteriorly extended posterior “basibranchials” (Figure 4). This is unusual among placoderms, though there are few comparative examples. Recently, Carr et al. (2009) attempted a revised interpretation of the gill skeleton of *Pseudopetalichthys* in light of *Cowralepis*. However, the additional details provided by *Paraplesiobatis* reveals that neither of their reconstructions is quite accurate.

A key difference in the gill skeletons of *Pseudopetalichthys* and *Paraplesiobatis* is that the gill skeleton of the former appears segmented between the “basibranchials” and the “ceratobranchials.” However, a similar pattern can be generated in *Paraplesiobatis* by embedding the reconstructed arches in a plane (Figure 4). The differences could, therefore, reflect differences in the degree of ossification or partial burial of the gill skeleton in *Pseudopetalichthys*.

KGM 1983/294 exhibits a broad, thin, spatulate ossification flanking the basihyal. Unusual broad plates in a similar position are observed in *Pseudopetalichthys* and interpreted by Gross (1962) as mandibular elements. However, in comparison with KGM 1983/294 and *Cowralepis* (based on Carr et al., 2009) we consider it reasonable to argue that these are, in fact, expanded ceratohyals. The resulting interpretation brings the gill skeletons of *Paraplesiobatis* and *Pseudopetalichthys* into close agreement.

### 4.2 Comparison with other placoderms

Other placoderms preserving branchial arch material include the arthrodires *Tapinosteus* (Stensiö, 1969) and *Cowralepis* (Ritchie, 2005); the rhenanids *Gemuendina* (Gross, 1963) and *Jagorina* (Stensiö, 1969); pytctodontids (Forey & Gardiner, 1986; Long, 1997; Miles, 1967) and the so‐called “stensioellids” *Stensioella* and *Pseudopetalichthys* (Gross, 1962), the latter will be treated in a separate subsection below. The remaining “stensioellids” will be treated elsewhere.

#### 4.2.1 Basihyal

**Figure 5:**
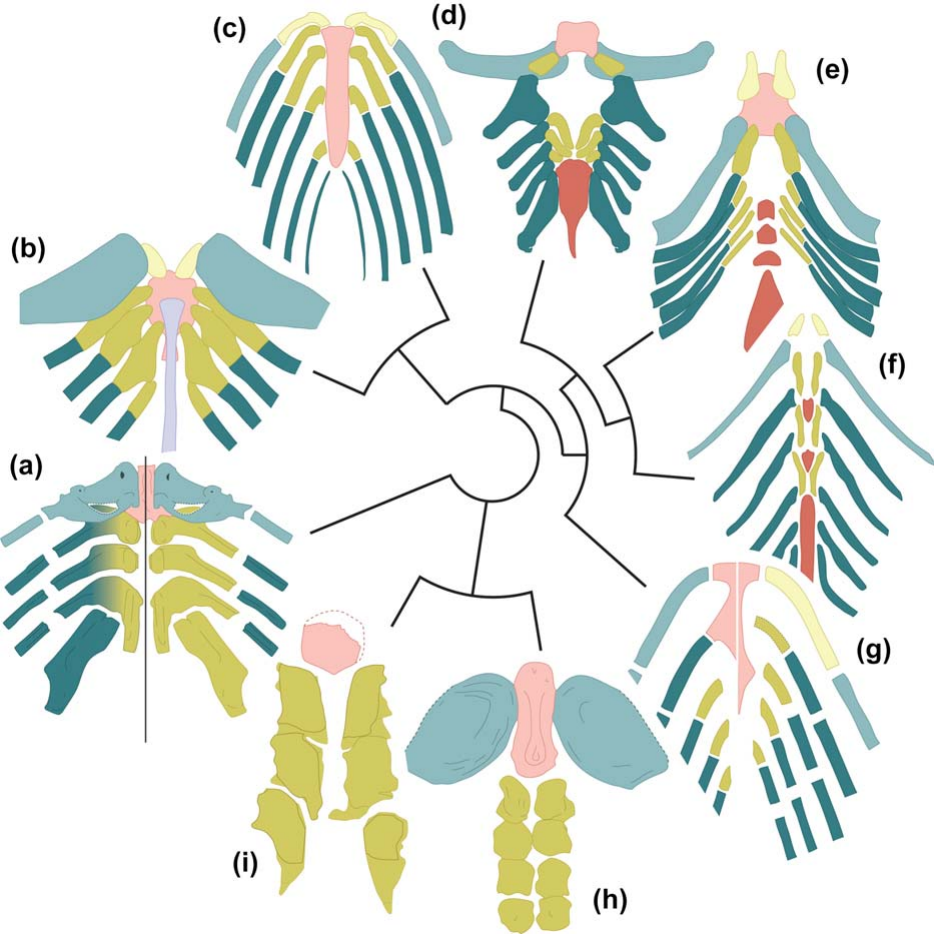
Phylogenetic comparison of ventral gill arch patterns in early jawed vertebrates. (a) Two possible interpretations of *Paraplesiobatis*. (b) *Glyptolepis* after Jarvik (1972). (c) *Mimipiscis* after Gardiner (1984). (d) *Scapanorhynchus* after Garman (1913). (e) *Debeerius* after Grogan and Lund (2000).(f) *Ozarcus* after Pradel et al. (2014). (g) *Acanthodes* after Miles (1973) (left), and Gardiner (1984) (right). (h) *Cowralepis* after Carr et al. (2009). (i) *Tapinosteus* after Stensiö (1969). Not to scale

A single median basihyal is common (possibly universal) among placoderms (Figure 5). This structure is observed clearly in *Tapinosteus* (Stensiö, 1969), *Cowralepis*, and *Pseudopetalichthys*. Gross (1963) describes a “copula element” in both direct examinations and radiographs of *Gemuendina*. Based on the position of this bone relative to the other pharyngeal elements, it is reasonable to conclude that it is also a basihyal bone. The basihyal of KGM 1983/294 resembles the morphology of *Cowralepis* in the structure of its longitudinal ventral keel. Such a keel appears to be absent in *Tapinosteus* (Stensiö, 1969).

#### 4.2.2 The number and structure of branchial arches in placoderms

In all examples of placoderms, and here further corroborated by KGM 1983/294, the basihyal is followed by a paired series of basibranchial‐like ossifications. In *Tapinosteus* (Figure 5; Stensiö, 1969) and *Cowralepis* (Figure 5; Carr et al., 2009), the first pair of “basibranchials” either articulates with or is very closely apposed to the posterior of the basihyal.

The branchial bones of KGM 1983/294 consist of arches comprising only two segments: a medial and lateral one. The area corresponding to the “basibranchials” (“hypobranchials” of Stensiö) in other placoderms are joined to extended lateral processes that would themselves correspond to either hypobranchials or ceratobranchials. The first arch is of uncertain hyoid or branchial identity and the fifth arch does not have its own “basibranchial” region, but rather joins the preceding (fourth) arch. The “basibranchial” series of *Cowralepis* numbers four elements (Carr et al., 2009), while there are three pairs in *Tapinosteus* (Stensiö, 1969). However, Stensiö reconstructs multiple arches joining these bones based on a chondrichthyan, and more specifically, hexanchiform, interpretive model.

In *Gemuendina*, Gross (1963) illustrates up to four separate arches. However, an indistinct fifth arch can be observed in his plate 8, figure B. Notably, the proximal end of the first arch bears a spatulate expansion (shown both in Gross's illustration and photographic plate). This spatulate expansion is nestled behind the mandibular arch cartilage and could reasonably be interpreted as a ceratohyal.

Based on these details, it seems reasonable to conclude that placoderms generally possess a median basibranchial and at least four “basibranchial” elements, corresponding to at least four individual pharyngeal arches. What remains unclear is whether the “basibranchial” elements should, in fact, be interpreted as that, or whether they more realistically correspond to hypobranchials of other gnathostomes.

### 4.3 Comparison with crown‐group gnathostomes

#### 4.3.1 Similarities with chondrichthyans

The presence of a median basihyal in *Paraplesiobatis* coupled with no apparent hypohyals compares well with modern chondrichthyans (Figure 5; see e.g., Carvalho et al., 2013). The outline of the basihyal in KGM 1983/294 is similar to that of the Carboniferous holocephalan *Debeerius* (Grogan & Lund, 2000) in being roughly trapezoidal with anterolateral “notches,” possibly accommodating articulation with the hyoid arch (Figure 5).

#### 4.3.2 Similarities with osteichthyans

Placoderm pharyngeal skeletons agree with osteichthyan examples in having enlarged/differentiated ceratohyals (Figure 5). KGM 1983/294 further resembles osteichthyans in having the last arch in the series join the penultimate arch. Nothing in the arches of KGM 1983/294 or any other placoderm example considered here can be interpreted as a posteriorly directed hypohyal.

### 4.4 Interpretation and reconstruction

*Paraplesiobatis* specimen KGM 1983/294 preserves at least five pharyngeal arches. Here, we offer two competing interpretations in relation to the arches of other gnathostomes (Figure 5). The first interpretation is that the first preserved arch is a hyoid arch (comprising a pair of ceratohyals articulating with the basihyal). The subsequent arches would therefore consist of four branchial arches. Alternatively, the model of the first arch is, in fact, a composite of two separate elements that were pushed together when the front of the gill skeleton was dislodged. The ovate, blade‐like element would correspond to a displaced ceratohyal, the upper rod‐shaped region would be the medial element of branchial arch 1. In this case, the arches correspond to branchial arches only, numbering 1–5. As in osteichthyans, arch 5 articulates with arch 4, but is highly differentiated.

The branchial architecture of *Paraplesiobatis* is not easily compared with other gnathostomes and, with the exception of the basihyal, standard nomenclatural terms are difficult to apply. The ventral aspect of the branchial skeleton conforms equivocally to nomenclatural conventions of gnathostome branchial arches. Furthermore there are no evident epibranchial or pharyngobranchial ossifications. The branchial basket appears to have met the lateral margins of the braincase directly, without any intervening ossifications. Alternatively, these elements were small, possibly unossified, and therefore unpreserved.

### 4.5 Phylogenetic distributions

Under the assumption that placoderms (including *Paraplesiobatis*) are stem‐group gnathostomes (Figure 5), we can make a number of phylogenetic inferences. There are assumed to be monophyletic chondrichthyan and osteichthyan crown groups. Most analyses favor placoderm paraphyly, however, King, Qiao, Lee, Zhu and Long (2016) recently demonstrate problems with this result and recover a majority of placoderms as monophyletic (King et al., 2016) under modified analytical methods. We, therefore, elect to make no prior assumptions about placoderm monophyly. Unless otherwise indicated, we will offer interpretations consistent with both monophyly or paraphyly of placoderms.

A median basihyal is apparently universal among placoderms, it is common to crown and some putative stem‐group chondrichthyans. The alternative state, a pair of hypohyals connecting to a median basihyal, appears to be unique to osteichthyans among extant gnathostomes.

When extinct gnathostomes are considered, *Acanthodes* raises a caveat with respect to the presence or absence of hypohyals in chondrichthyans. This taxon is now increasingly considered a stem‐group chondrichthyan (Brazeau & de Winter, 2015; Giles, Friedman, & Brazeau, 2015; Zhu et al., 2013), but it presents a pattern of pharyngeal segmentation with multiple (not necessarily competing) interpretations. The ceratohyal of *Acanthodes*, according to Gardiner (1984) is composed of two discrete ossifications. This could be interpreted as either a subdivided ceratohyal or a ceratohyal and a hypohyal. Furthermore, nothing precludes interpretation of the osteichthyan hypohyal as a subdivision of the ceratohyal. However, the heavily subdivided visceral and branchial series of *Acanthodes* may be apomorphic, as there is no evidence of this type of ossification pattern in other non‐acanthodiform acanthodians (Blais, Hermus, & Wilson, 2015; Brazeau, 2012; Burrow, Davidson, den Blaauwen, & Newman, 2015; Hanke, Davis, & Wilson, 2001; Hanke & Wilson, 2010).

The absence of a median basihyal in *Ozarcus* (Pradel et al., 2014) is anomalous and can be considered a derived state, either of that taxon or symmoriiforms more generally. Paired hypohyals in *Ozarcus* resemble osteichthyans, but their phylogenetic significance is somewhat more ambiguous (Figure 5). Hypohyals also observed in the stem‐holocephalan *Debeerius ellefseni* (Grogan & Lund, 2000). They may have been gained in prior to the origin of the gnathostome crown and lost (at least) twice in chondrichthyans (minimum three steps). Alternatively, they were gained in symmoriiforms, osteichthyans, and *Debeerius* (also three steps). A more parsimonious distribution for these structures could be arrived at by placing *Ozarcus* (along with other symmoriiforms) on the holocephalan stem (Figure 5), as has been suggested elsewhere on the basis of other lines of evidence (Coates, Gess, Finarelli, Criswell, & Tietjen, 2017; Coates & Sequeira, 2001; Giles et al., 2015).

Median, unpaired basibranchials are absent in placoderms. No examples have been identified in *Acanthodes*, the only acanthodian for which substantial gill skeleton ossifications are known. The chain of median basibranchials identified by Nelson (1968) are disputed by Miles (1973) and Gardiner (Gardiner, 1984). Computed tomography investigations of two other acanthodian species is currently underway by the authors and RP Dearden (Imperial College London) will help clarify this. However, provisionally, we argue that the absence of unpaired median mineralisations posterior to the basihyal is primitive and that their origins in osteichthyans and chondrichthyans are separate. Alternatively, the basibranchial of osteichthyans is homologous to the basihyal of chondrichthyans, but has become foreshortened in elasmobranchs.

## AUTHOR CONTRIBUTIONS

MDB and MF designed the original study; MDB wrote the manuscript with input from MF. MDB and AJ performed the segmentation. RA conducted the synchrotron scanning and tomography reconstructions with assistance from MF and MDB. MDB and AJ composed the figures.

## Supporting information

Supporting Information

Supporting Information Figure 1
