# Supplementary material for: A three‐dimensional placoderm (stem‐group gnathostome) pharyngeal skeleton and its implications for primitive gnathostome pharyngeal architecture
Source: J Morphol. 2017 May 23;278(9):1220–8. doi: 10.1002/jmor.20706 (PMC5575467; doi:10.1002/jmor.20706)
Supplement: Supplementary file 1 — Supporting Information [file JMOR-278-1220-s001.docx]

Supporting Information Figure 1. Illustration of gray level variation and artefacts of tomography scanning in *Paraplesiobatis* specimen KGM 1983/294. Coronal tomography image showing longitudinal axis of ring artefacts (A). Plot of grayscale variation over the length of the scan (X axis corresponds to vertical line in A; numbers correspond to numbered yellow wedges in same panel), showing abrupt renormalization of gray values (B).
